# Effects of platelet-rich plasma in a model of bovine endometrial inflammation *in vitro*

**DOI:** 10.1186/s12958-016-0195-4

**Published:** 2016-09-13

**Authors:** Maria Giovanna Marini, Claudia Perrini, Paola Esposti, Bruna Corradetti, Davide Bizzaro, Pietro Riccaboni, Eleonora Fantinato, Giuseppe Urbani, Giorgio Gelati, Fausto Cremonesi, Anna Lange-Consiglio

**Affiliations:** 1 Department of Life and Environmental Sciences, Università Politecnica delle Marche, Ancona, Italy; 2 Large Animal Hospital, Reproduction Unit, Università degli Studi di Milano, Via dell’Università 6, 26900 Lodi, Italy; 3 Large Animal Hospital, Anatomo-Pathology Unit, Università degli Studi di Milano, Lodi, Italy; 4 Private Practitioner, Milano, Italy

**Keywords:** Cattle, Endometrial cells, Platelet-rich plasma, LPS, gene expression

## Abstract

**Background:**

Endometritis reduces fertility and is responsible for major economic losses in beef and dairy industries. The aim of this study was to evaluate an alternative therapy using platelet-rich plasma (PRP). PRP was tested *in vivo*, after bovine intrauterine administration, and *in vitro* on endometrial cells.

**Methods:**

Bovine endometrial cells were cultured until passage (P) 10 with 5 % or 10 % PRP. Effect of PRP on endometrial cell proliferation and on the expression of genes [prostaglandin-endoperoxide synthase 2 (*COX2*), tumor protein p53 (*TP53*), oestrogen receptors (*ER-α* and *ER-β*), progesterone receptor (*PR*) and *c-Myc*] involved in the regulation of oestrus cycle and fetal-maternal interaction were evaluated. Moreover, to evaluate the ability of PRP to counteract inflammation, 10 and 100 ng/ml of bacterial endotoxin lipopolysaccharide (LPS) were used to inflame endometrial cells *in vitro* for 1, 6, 12, 24 and 48 h. The expression of genes such as interleukin 1β (*IL-1β*), interleukin-8 (*IL-8*), inducible nitric oxide synthase (*iNOS*), prostaglandin-endoperoxide synthase 2 (*COX2/PTGS2*), and the release of PGE-2, IL-1β and IL-8 were evaluated.

**Results:**

*In vivo* treatment with PRP increased the detection of PR. *In vitro*, 5 % PRP at passage 5 increased proliferation rate and induced a significant increase in the expression of all studied genes. Furthermore, the results revealed that 10 ng/ml of LPS is the most effective dose to obtain an inflammatory response, and that PRP treatment significantly down regulated the expression of pro-inflammatory genes.

**Conclusion:**

This study lays the foundations for the potential treatment of endometritis with PRP *in vivo*.

## Background

The wall of the uterus consists of endometrium, myometrium and perimetrium (tunica serosa). The bovine endometrium is a complex tissue that comprises the tunica mucosa (lamina epithelialis and lamina propria consisting of loose connective tissue) and the tunica submucosa of the uterus. In the lamina propria, neutrophils and lymphocytes are commonly detectable. The endometritis is characterized by an increase number of inflammatory cells associated to epithelial erosion and/or necrosis and diffuse oedema of endometrium [1]. Meta-analysis studies show that endometritis reduces pregnancy rate by 16 % [2]. The economic losses related to this disease are substantial and associated with a great impact on the economy to drop in milk yield and calves, and treatment costs [3]. Approximately, 80 % to 100 % of cows have intrauterine bacterial contamination in the first two weeks postpartum. In most cases, clinical disease does not develop, as the normal uterus is able to clear a bacterial infection efficiently [4]. In 10 %-20 % of cows, however, the infection is not controlled and may lead to chronic uterine inflammation [1, 5]. It is commonly accepted that the development of uterine infection depends on the immune response of the cow, as well as species and number of bacteria [1]. Economically, important uterine diseases in cattle are commonly associated with bacterial infection by *Escherichia coli*, *Trueperella pyogenes*, *Fusobacterium necrophorum* or *Prevotella* species [1, 5–8]. The most significant pathogenic bacteria responsible for uterine infection are *E. coli*, which produce an endotoxin lipopolysaccharide (LPS) that is present in their cell wall.

Establishment of uterine bacterial infection may also depend on metabolic disease, although the specific mechanisms are still not clear or by endocrine environment, that affects the likelihood of bacteria elimination [9–11].

The inflammatory response is a complex process involving many signalling cascades. In the genital tract, the initial response of the endometrium against infection is dependent on innate immunity and mucosal defence systems [3, 12]. The uterine immune response is provided at the cellular level, by uterine leukocytes and polymorphonuclear cells that are the cells phagocytizing and clearing bacteria [13]. In horse, at the molecular level, cytokines have a significant role in the recruitment of inflammatory cells [14]. In cattle, interleukin-6 and TNF-α stimulate the production of antimicrobial peptides that assist in the elimination of pathogenic bacteria [15, 16]. Moreover, bovine endometrial epithelial and stromal cells can respond to bacterial LPS through the Toll-like receptor (TLRs) [17]. Activated TLRs subsequently stimulate the production of pro-inflammatory cytokines and chemokines [18].

Literature on the treatment of endometritis in cattle is extensive and at times inconsistent. Specifically, uterine disease treatments aim at reverting inflammatory changes that impair fertility, whilst enhancing uterine defence and repair [19]. In cows, many therapeutic agents and procedures have been developed to treat endometritis, including systemic or intrauterine administration of antibiotics [20–22], or administration of PGF2α and its analogue [20–24]. The intrauterine infusion of antiseptics (e.i. Lugol’s solution) has been tested although side-effects on future fertility of the cows have been reported [23, 25]. Furthermore, approaches using proteolytic enzymes [21], administration of estradiol [26, 27] or GnRH [28] have also been described. Even if all these treatments show some effects on uterine healing, no definitive results can be extrapolated from published clinical trials.

Platelet-rich plasma (PRP) is an emerging therapeutic application in tissue regeneration and engineering due to its enrichment in growth factors with mitogenic and anti-inflammatory potential [29, 30]. In particular, PRP is a concentration of platelets (3-5 fold the plasma baseline level) containing transforming growth factor β1 (TGF-β1) and TGF-β2, platelet derived growth factors (PDGF-AA, PDGF-BB, PDGF-AB), insulin-like growth factor 1 (IGF-I), epidermal growth factor (EGF), vascular endothelial growth factor (VEGF), fibroblast growth factor (FGF) and hepatocyte growth factor (HGF) that are very important for regeneration processes. Indeed, these growth factors act synergistically to increase the infiltration of neutrophils and macrophages, to promote angiogenesis, fibroplasia, matrix deposition and, ultimately, re-epithelialization, inducing the consequent tissue regeneration [29]. Lastly, the presence of anti-inflammatory molecules, including HGF [31] also confers on PRP the ability to suppress inflammatory process.

In this context, the specific aim of this study was to investigate the effect of PRP *in vivo* and *in vitro*, on a model of healthy or LPS stressed bovine endometrial cells. *In vivo*, the progesterone receptor (PR) expression was detected immunohistochemically. The steroid hormone progesterone (P4) plays a key role in reproductive events associated with establishment and maintenance of pregnancy. Conceptus growth and development require the action of P4 on the uterus to regulate endometrial function, including conceptus-maternal interactions, pregnancy recognition, and uterine receptivity to implantation [32]. Indeed, low P4 concentrations have been implicated as a causative factor in the low pregnancy rates observed in high-yielding dairy cows [33]. *In vitro*, genes involved in the regulation of oestrous cycle, in pro-inflammatory process and the release of some cytokines were evaluated.

## Methods

### Experimental design

This study was based on three experiments. The first experiment evaluated the endometrium histologically and immunohistochemically, after *in vivo* PRP administration, using PR receptor as a marker of cell receptivity. The second experiment evaluated the effect of a 5 % and 10 % concentration of PRP in culture medium, on *in vitro* endometrial cell proliferation and on the expression of some genes involved in the regulation of oestrous cycle and fetal-maternal interaction, to establish whether it is able to improve the functions of this cell line. The genes included prostaglandin-endoperoxide synthase 2 (*COX2* or *PTGS2*), tumor protein p53 (*TP53*), oestrogen receptors (*ER-α* and *ER-β*), progesterone receptor (*PR*) and v-myc avian myelocytomatosis viral oncogene homolog (*c-Myc*). The third *in vitro* experiment evaluated the ability of PRP to counteract an *in vitro* model of inflammation by stressing endometrial cells with LPS at different times and concentrations. Expression of pro-inflammatory genes and release of some cytokines were evaluated.

### Materials

Chemicals were obtained from Sigma-Aldrich Chemical (Milan, Italy) unless otherwise specified. LPS was purchase by Sigma-Aldrich Chemical (E. coli 0:111B4; L2630 catalog number). Tissue culture plastic dishes were purchased from Euroclone (Milan, Italy).

#### Animals

All procedures were performed according to approved animal care and use protocols of the institutional ethics committee and to good veterinary practice for animal welfare as to European directive 2010/63/UE. Written farmers’ consent was obtained at the beginning of the study.

From a group of Holstein Friesian, animals at 150-180 days in milking belonging to a 180 cows dairy farm located in North Italy, 14 cows bearing a well-developed corpus luteum (CL) diagnosed by B-mode ultrasound evaluation of the ovaries, were selected. They received an i.m. luteolytic dose of PGF2α to synchronize the estrous cycle. All animals (*n* = 10) showed estrous signs in the following 96 hours and at that time bacteriological and cytological analyses, trans rectal and vaginal palpation, and sonography of the reproductive tract were conducted to exclude genital diseases. These animals were enrolled for the first study.

Endometrial samples for *in vitro* study were collected from slaughtered bovines under legal regulations

### Preparation of platelet-rich plasma

#### Collection of blood

Blood was obtained from two donor cows at forty days in milking, as this is the period the circulating platelet count is higher than other periods (data not shown). These animals were in good health, free from infectious diseases and they did not receive medication during the previous two months. The collection of blood and the preparation of PRP, with the method of double centrifugation, were performed as reported by Lange-Consiglio et al. [34]. After surgical scrub preparation of a few centimeters of skin around the subcutaneous mammary vein, 450 ml of blood was collected in *ad hoc* Terumo blood bags (Terumo Srl, Rome, Italy) containing citrate-phosphate-dextrose-adenine (CPDA-1) using the 16-gauge needle provided with the bags. The bags were transported at 4 °C to the laboratory within 2 h of collection and immediately processed.

#### Double centrifugation method

All separation steps were performed under a horizontal laminar flow hood in aseptic conditions. To prepare the PRP, the blood was drawn into sterile Falcon tubes of 50 mL each (EuroClone SpA, Milan, Italy). The tubes were centrifuged at 100 x g for 30 min at 4 °C. This caused separation of the blood into three components: red blood cells at the lowest level, “buffy coat” in the middle layer, and platelet rich plasma (PRP) in the upper layer. Afterward, the PRP was carefully aspirated and distributed in new 50-ml tubes and centrifuged again at 1,500 x g for 10 min at 4 °C to obtain the platelet pellet and the poor platelet plasma (PPP) on the upper layer. Afterward, two-thirds of the volume of PPP was aspirated for later use and the pellet mixed in the residual PPP volume to allow for platelet count before the final dilution with PPP to obtain PRP at a standard concentration of 1 × 10^9^ platelets/ml [34]. All platelet counts on peripheral blood and PRP were performed using a HeCo Vet automatic hematology analyzer (SEAC, Florence, Italy). The total amount of PRP obtained for each donor was aliquoted in 10 ml ready-to-use doses that were stored in syringes. The syringes were then frozen at −80 °C and thawed at 37 °C three times [35] to allow the release of platelet-derived factors. The PRP was subjected to aerobic and anaerobic bacteriological examination to verify its sterility. Syringes containing the PRP dose were kept frozen at −20 °C until use.

### Experiment 1: *in vivo* effect of intrauterine administration of PRP

At day 4 post estrus, all previously selected animals were checked by ultrasound for the presence of a newly formed CL and were randomly divided in two groups. Seven cows were treated with PRP while the other seven animals were enrolled as control (CTR). Physiological solution (0.9 % NaCl) was used as placebo for these latter. Ten ml of PRP or ten ml of physiological solution were aseptically infused into the uterus by a disposable sterile catheter included in a protective sheath guided into the cervix by manipulation per rectum. To prevent transfer of vaginal flora into the uterine lumen, the protective sheath was removed just after cervix penetration and the catheter was hence introduced into corpus uteri where PRP was infused.

#### Endometrial biopsy

Endometrial biopsies were collected using a stainless steel Hauptner equine endometrial biopsy instrument as previously described by Chapwanya et al. [15] and Katagiri and Takahashi [36]. Samples were obtained just before treatment at day 4 post estrus (T0) and 7-day post treatment, that is at day 11 post estrus (T1). Briefly, after cleaning the base of tail, perineum and external genitalia, animals were given caudal epidural anesthesia (4–5 ml 2 % of lidocaine). Afterwards, the biopsy instrument included in a protective sheath was introduced into the uterus through the cervix by manipulation per rectum. Once the cervix was passed, and the protective sheath ruptured, the tip of the biopsy instrument inside the uterus was identified using the hand per rectum. Then, with the help of the hand in the rectum, the medial uterine wall was gently pressed into the instrument jaws and biopsies were taken from the dorsomedial aspect of one horn just anterior to the bifurcation. Biopsies for histology were gently removed from the biopsy instrument with a fine gauge needle and transferred to a vial of fixation solution. All biopsies were processed by the same operator. The samples were performed for histological and immunocytochemical studies.

#### Histological and immunohistochemical examinations

Samples were routinely formalin-fixed (10 % buffered formalin) for 48-72 hours and paraffin-wax embedded. Four-micrometer thick sections were stained with hematoxylin and eosin (HE), while others were prepared on poly-L-lysine–coated glass slides for immunohistochemistry to detect the presence of PR.

The immunohistochemical staining of all samples was performed using the avidin–biotin–peroxidase complex procedure with a commercial immunoperoxidase kit (Vectastain Standard Elite; Vector Laboratories, Inc., Burlingame, CA, USA).

Tissue sections were immersed in a pre-heated solution at 94 °C of Dewax and HIER Buffer H (Thermo Fischer Scientific, Lab Vision Corporation, Fremont, CA, USA) diluted 1:15 with deionized water for 40 minutes. This solution is designed to simultaneously dewax and perform heat induced epitope retrieval.

Endogenous peroxidase was blocked using 1 % hydrogen peroxide in Tris buffer for 45 min.

Sections were incubated for 18 hours at 4 °C with anti-PR (Thermo Fischer Scientific, Lab Vision Corporation, Fremont, CA, USA) mouse monoclonal antibodies diluted 1:400. After incubation with the secondary biotinylated anti-mouse immunoglobulin (diluted 1:200; Vector Laboratories, Inc.) for 30 min, the avidin–biotin–peroxidase complex method (Vector Laboratories, Inc.) was performed. Positive staining was visualized with 3.3-diaminobenzidine-4 HCl (Vectastain, Vector Laboratories, SK-4100) and nuclei were counterstained with Mayer’s hematoxylin. Diluent negative control sections were produced by omission of the primary antibody.

#### Evaluation of histological and immunohistochemical data

Tissue samples were evaluated in order to assess the absence of pathological lesions.

The immunohistochemically stained tissue slides were examined using standard light microscopy. Three randomly selected areas were evaluated per section. The staining results were independently scored semi quantitatively by different blinded operators at 400X magnification. The results of PR protein expression were assessed by categorizing immunoreaction of epithelial cells, muscular layer and glandular cells into five groups according to the percentage of positive cells. These group were: 0) no positive cells; 1) <1 % of positive cells; 2) from 1 % to 10 %; 3) from 11 % to 33 %; 4) from 34 % to 66 % and 5) from 67 % to 100 % [37]. The intensity of labeling was graded as: weak positive staining (W); moderate positive staining (M); intense positive staining (I) and scored as follows: W = 1, M = 2, I = 3. A total score was conferred adding together the positive cells percentage score and the intensity of immunolabeling score [37].

### Experiment 2: effect of different concentrations of PRP on *in vitro* endometrial cells proliferation and gene expression

#### Tissue collection and cell isolation

Fresh bovine uteri were collected from three different cows at the slaughterhouse intended for human consumption and unrelated to our experiments. Samples were obtained from healty normal-cycling cows at diestrus stage (middle-late luteal phase). Only uteri belonging to cows with an obvious corpus luteum on the ovary were used for endometrial fragment collection and ensuing cell culture.

Endometrial samples were kept at 4 °C in saline solution supplemented with 4 μg/ml amphotericin B, 100 IU/ml penicillin-100 μg/ml streptomycin and processed within 2 h. Endometrial stromal cells were obtained according to the protocol described by Donofrio et al., [38]. Briefly, the endometrium was digested in 25 ml of sterile filtered Hanks’ buffered salt solution supplemented with 50 mg collagenase II, 100 mg bovine serum albumin, and 10 mg DNase I for 90 minutes at 38.5 °C in a shaking bath. Then, cells were filtered with an 80-μm filter, centrifuged at 300 x g for 10 minutes, and washed twice in PBS. Before seeding, the total number of viable cells was evaluated by the exclusion method staining with trypan blue and using a Bürker chamber.

#### Cells expansion

A pool of endometrial stromal cells cultures from three different cows was established in Dulbecco's Modified Eagle's Medium high glucose (HG-DMEM) supplemented with 10 % FBS, 100 UI/ml penicillin–100 μg/ml streptomycin, 0.25 mg/ml amphotericin B and 2 mM L-glutamine (standard complete medium). For maintenance of cultures, cells were plated in 75 cm^2^ flasks (SPL Cell culture flask 70025) at up to 1x10^5^ cells/cm^2^ and incubated at 38.5 °C with 5 % CO_2_ in a humidified atmosphere. To remove non-adherent cells, the medium was replaced for the first time after 48 h. Adherent cells were detached with 0.05 % trypsin-EDTA just prior to reaching confluence (80 %) and then reseeded for culture maintenance at the density of 1x10^4^ cells/cm^2^.

#### Proliferation studies

All data are representative of three independent experiments.

##### Doubling time (DT)

DT for passages 1–10 (P1 and P10) was assessed plating 9x10^3^ cells into six-well tissue culture plates. Every 4 days cells were trypsinized, counted and reseeded at the same density. Mean DT was calculated from day 0 to day 4. The DT value was obtained for each passage according to the formula DT = CT/CD, where CT represents the culture time and CD = log (Nc/No)/log_2_ represents the number of cell generations (Nc represents the number of cells at confluence, No represents the number of seeded cells).

##### Growth curves

To obtain growth curves at P1, P5 and P10 endometrial cells were plated at the density of 9x10^3^ cells into six-well tissue culture plates. Every two days, over the 12 days of culture, cells from one well of each plate were detached and counted.

#### In vitro effect of PRP on endometrial gene expression

In this study, culture medium was replaced for the first time after 48 h to remove non-adherent cells and changed with complete standard medium supplemented with two different concentrations of PRP, 5 % and 10 %, as a FBS substitute. Control cells were grown in complete standard medium supplemented with 10 % FBS. The cells were expanded for 10 passages, which was the last time point included in our study. Cells from each well were then collected to evaluate mRNA expression of genes involved in the regulation of oestrous cycle and fetal-maternal interaction by quantitative real-time PCR. The genes evaluated were prostaglandin-endoperoxide synthase 2 (*COX2*), tumor protein p53 (*TP53*), oestrogen receptors (*ER-α* and *ER-β*) and progesterone receptor (*PR*). The expression of *c-Myc* was also evaluated.

Molecular characterization of endometrial cells was performed at passages 1, 3, 5 and 10 by qualitative PCR. Data were obtained from three replicates.

### Experiment 3: *In vitro* effect of PRP after LPS treatment on gene expression

P5 endometrial cells were plated in six well plates at a density of 50,000 cells/well in DMEM standard complete medium containing two different concentration of LPS, 10 ng/ml and 100 ng/ml. Evaluation of the expression of genes involved in inflammation was performed at 1, 6, 12, 24 and 48 h. The LPS concentrations were chosen on the base of results of Herath et al. [39] that demonstrated level of LPS around 100 ng/ml in cows with clinical endometritis. Supernatants were collected and stored at -20 °C until being used for ELISA to measure PGE-2, IL-1β and IL-8.

To study the *in vitro* effect of PRP in cells inflammed with LPS, endometrial cells were treated with 10 ng/ml LPS and 5 % PRP for 1, 6, 12, 24 and 48 h. At each time point, the expression of same genes was analyzed. Data were obtained from three replicates.

#### Molecular characterization

Total RNA was extracted from endometrial cells using TRI Reagent Solution (Life Technologies, Monza, Italy). Samples were then treated with DNase in order to eliminate DNA contamination. RNA concentration and purity were measured by Nanodrop Spectrophotometry (NanodropH ND1000). The quality and integrity of the total RNA extracted were verified by electrophoresis on a 1.5 % (w/v) agarose gel. The cDNA was synthesized from total RNA (200 ng) using the PrimeScript RT reagent Kit (Takara Bio). Gene expression evaluation was performed using bovine specific sequences. Oligonucleotide primers were designed using open source PerlPrimer software v. 1.1.17 based on available NCBI *Bos taurus* sequences or on mammal multi-aligned sequences. Primers were designed across an exon–exon junction in order to eliminate genomic DNA amplification and their sequence conditions and the references used are shown in Table 1.

**Table 1:** Oligonucleotide sequences used for RT-PCR analysis

Marker	Forward 5'-3'	Reverse 5'-3'	T annealing	bp
Interleukin-1β (IL-1β)	TGCAGCTGGAGGAAGTAGAC	GTCGGGCATGGATCAGACAA	60	338
Interleukin 8 (IL-8)	ACATACCCTGCCACAAGGC	TGGGGACTGCTCTTCCCTCT	57	146
Inducible nitric oxide synthase (iNOS)	GGACCTCAACAAAGCCCTGA	CCTTGACCCAATAGCTGCCA	60	293
Prostaglandin-endoperoxide synthase 2 (COX2)	CATGGGTGTGAAAGGGAGGAA	AAAGACGTCAGGCAGAAGGG	60	700
Tumor protein p53 (TP53)	CCTAGGAGCACTAAGCGAGC	GCCCCTCTCTCTTGAGCATT	55	268
Oestrogen receptor alpha (ERα)	AGGGAAGCTCCTATTTGCTCC	CGGTGGATGTGGTCCTTCTCT	55	234
Oestrogen receptor beta (ERβ)	GCTTCGTGGAGCTCAGCCTG	AGGATCATGGCCTTGACACAG	55	262
Progesterone receptor (PR)	GAGAGCTCATCAAGGCAATTGG	CACCATCCCTGCCAATATCTTG	55	227
v-myc avian myelocytomatosis viral oncogene homolog (c-Myc)	GCGCCGCATTCGCGAAACTT	TGAGGGGCATCGCTGCAAGC	58 °C	214
Glyceraldehyde-3-phosphate dehydrogenase (GAPDH)	ATGAGATCAAGAAGGTGGTG	CCAAATTCATTGTCGTACCAG	60	190

Qualitative PCR analysis was performed in a 25 μl final volume with Taq DNA Polymerase recombinant commercial kit (Invitrogen Life Technologies) under the following conditions: initial denaturation at 94 °C for 2 minutes, 32 cycles at 94 °C for 30 seconds (denaturation), 55–60 °C for 30 seconds (annealing), 72 °C for 30 seconds (elongation) and final elongation at 72 °C for 10 minutes. For conventional PCR, primers were used at 200 nM final concentrations.

Quantitative PCRs were performed with SYBR green method in a MyiQ iCycler thermal cycler (Biorad). PCR reactions were carried out in triplicate for each sample. The reactions were set on a strip in a final volume of 25 μl by mixing, for each sample, 1 μl of cDNA, 12.5 μl of 2X concentrated SYBR Premix Ex Taq II (Takara Bio) containing SYBR Green as a fluorescent intercalating agent, 0.2 μg forward primer, 0.2 μg of reverse primer and MQ water. PCR efficiencies were tested and found to be close to 1. The thermal profile for all reactions was 30 seconds at 95 °C and then 40 cycles of 5 seconds at 95 °C, 30 seconds at 60 °C. Fluorescence monitoring occurred at the end of each cycle. Efficiency of amplification for each primer was monitored through the analysis of serial dilution. Additional dissociation curve analysis was performed and in all cases showed a single peak. The data thus obtained were analyzed using the iQ5 optical system software version 2.0 (BioRad). The expression of each gene was normalized to the reference gene glyceraldehyde-3-phosphate dehydrogenase (*GAPDH*) (internal control) in order to standardize the results by eliminating variation in cDNA quantity. Such gene was identified among others used as endogenous control genes based on previous studies focusing on endometrial gene expression [40]. Relative gene expressions were presented with the 2^−ΔΔCt^ method [41].

#### Protein release by ELISA

Protein release (PGE-2, IL-1β and IL-8) was measured in cell-free supernatants obtained by centrifugation at 250 x g for 5 min and stored at −80 °C until measurement. Protein production was assessed according to the manufacturers’ instructions (Bovine PGE-2 MBS737103, MyBioSource; Bovine IL-1β Screening Set ESS0027, Thermo Fisher Scientific; CXCL8/IL-8 DuoSet DY208, R&D Systems). The Human CXCL8/IL-8 DuoSet was previously validated for measurement of bovine IL-8 [42]. The limits of detection for PGE-2, IL-ß and IL-8 were 1.0, 20.1 and 14.3 pg/ml, respectively.

Results are expressed in pg/ml.

### Statistical analysis

Statistical analysis was performed using GraphPad Instat Software version 3.00 for Windows (La Jolla, CA, US). For proliferation data, three replicates for each experiment were performed. Results are reported as mean ± standard deviation (SD). One-way analysis of variance (ANOVA) for multiple comparisons by Student-Newman-Keuls multiple comparison tests was used.

Immunohistochemical data were analyzed by unpaired Student's *t*-test.

For cytokines, statistical differences were determined using ANOVA followed by Dunnett's multiple comparison test, the Tukey–Kramer multiple comparisons test or unpaired t test.

Differences were considered statistically significant for *P* values < 0.05.

For quantitative PCR data, non-parametric tests were used. The Mann-Whitney U-test was employed to compare two groups (treated vs untreated). Results were considered significant if the value of *P* was < 0.01.

## Results

### Experiment 1: *in vivo* effectiveness of intrauterine administration of PRP

#### Histological examination

No pathological alterations were detected in any of the examined biopsies. All the samples were covered by a columnar simple epithelial monolayer. A moderately large amount of uterine glands was dispersed in the stroma. Scattered rare lymphocytes were also detectable in the stroma. Neither fibrosis nor hemorrhages were present.

#### Immunohistochemical evaluation

Positive nuclei to PR were brown-stained, while negative nuclei were blue stained by hematoxylin counterstain. Diluent negative controls showed no immunoreaction.

The PR immunoreactivity was evaluated separately in the surface epithelial cells, glandular epithelium and smooth muscle cells. The total scores are listed in Table 2.

**Table 2:** Total score for PGR expression in bovine according to the surface epithelial cells, glandular epithelium and smooth muscle cells

		Surface epithelium		Glandular epithelium		Smooth muscle	
	Subject	T0	T1	T0	T1	T0	T1
SALINE CTR	2	5	3.5	8	5	3	4.5
	4	8	4	8	8	6.5	6.5
	8	3	4	3	8	4	7.5
	9	8	7.5	7.5	8	5.5	7.5
	10	3	2	4	4	4	7
	11	4	4	5	4	5	4.5
	14	4	4	6	7	6	4
Average score		5.0±2.2^a^	4.1±1.7^a^	5.9±2.0^a^	6.3±1.9^a^	4.4±1.1^a^	5.8±1.66^a^
PRP	1	5	3.5	5.5	6.5	4	7.5
	3	3.5	0	4.5	7	2.5	6.5
	5	2	6.5	3.5	8	2.5	7
	6	8	8	7.5	8	8	7.5
	7	7.5	8	8	8	6	6.5
	12	5	5	6	7	3	7
	13	4	6	5	7	5	6.5
Average score		5.0±2.1^a^	5.3±2.8^a^	5.7±1.6^a^	7.4±0.6^b^	4.4±2^a^	6.9±0.4^b^

Legend: average score represent mean ± standard deviation. Different small letters superscripts (a,b) indicate statistically different comparisons (*P*<0.05) between T0 and T1

**Fig. 1:**
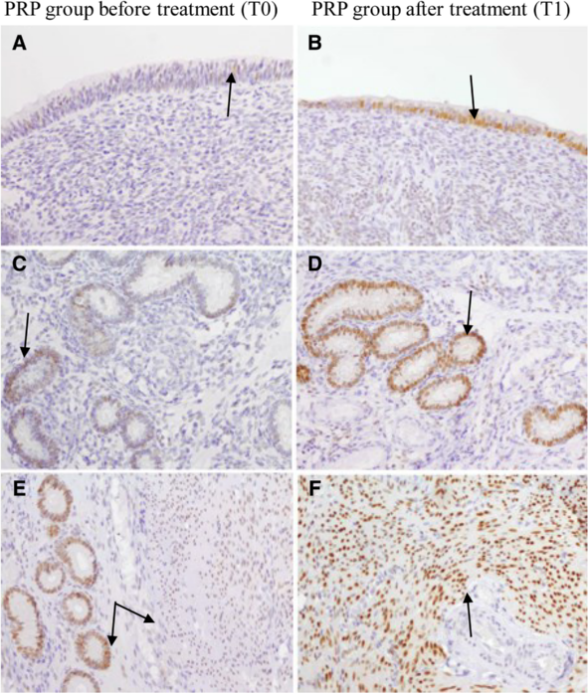
Immunohistochemical PR expression in uterine biopsies (20x, Haematoxylin counterstain). Figures in the left column are of PRP group treated at T0 while in the right column are of PRP group treated at T1. Arrows show the following results. Uterine surface epithelium showing: (**a**) weak positivity involving scattered cells (score W1); (**b**) and marked positivity in the majority of the epithelial cells (score I5). Glandular epithelium with (**c**) weak and diffuse immunostaining (score W4); (**d**) intense and diffuse imunostaining scored as I5. Smooth muscle cells with (**e**) moderate and diffuse immunolabeling (score M5). The same score is detectable in the close glandular epithelium. (**f**) Intense and diffuse immunohistochemical expression in smooth muscle cells (score I5)

PR expression has been detected in all these uterine compartments (Fig. 1). Treated animals in T1 showed the highest PR receptor score in glandular epithelium and smooth muscle cells compared to T0 (*P* < 0.05). In T1 control animals, glandular epithelium and smooth muscle cells obtained the same high average score that is not statistically different compared to T0 (*P* > 0.05).

### Experiment 2: effect of different percentages of PRP on *in vitro* endometrial cells proliferation and gene expression

#### Cells isolation and expansion

**Fig. 2:**
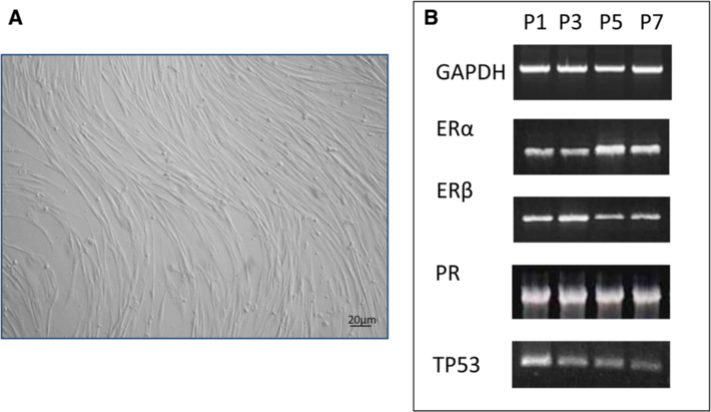
(**a**) Cell morphology. Monolayer of endometrial cells. Scale bar: 20 μM. Magnification 20X. (**b**) Molecular characterization of endometrial cells at diestrus stage. Qualitative PCR analysis for the expression of genes that influencing pre-implantation conceptus development (*ERα, ERβ* and *PR*)

Cells were selected on their ability to adhere to plastic. For endometrial cells, initial viability was >80 %. Cells displayed the typical fibroblast-like morphology (Fig. 2a). The molecular biology study on the isolated cells confirmed their endometrial nature due to the expression of *ER-α*, *ER-β*, *PR* and *TP53* (Fig. 2b).

#### Proliferation studies

**Fig. 3:**
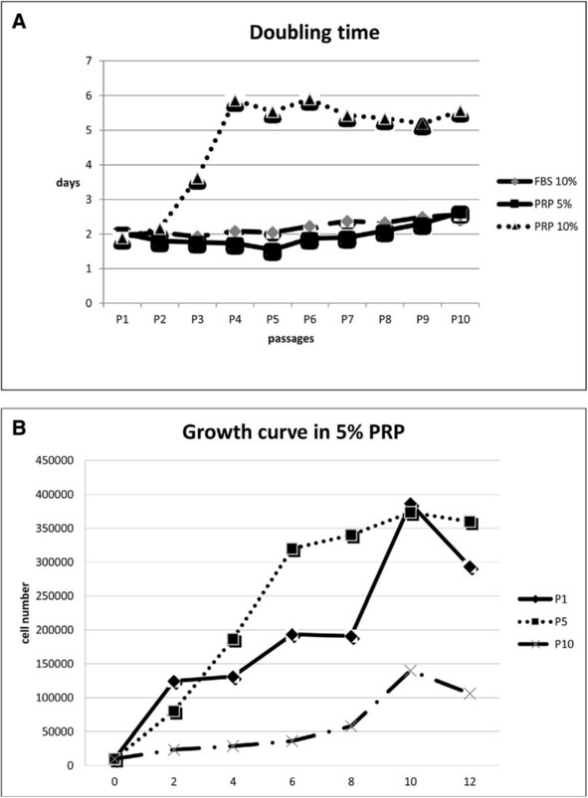
Proliferation studies. (**a**) Doubling time at different passages during cell culture in different culture conditions (5 % PRP and 10 % FBS). (**b**) Growth curve at P1, P5 and P10 for endometrial cells cultured with 5 % PRP

The DT value at P1 and P2 were similar between the cells grown in presence of 5 % PRP, 10 % PRP and 10 % FBS. After P2, for endometrial cells grown in presence of 5 % PRP, the proliferative ability increased until P5 while for cells grown in presence of 10 % PRP, the proliferative ability decreased and then remained constant until P10. No difference in the DT value for endometrial cells grown in presence of 10 % FBS were found over the passages studied (Fig. 3a). The mean DT value for endometrial cells grown in presence of 5 % PRP was 1.96 ± 0.31 days, while it was 4.64 ± 1.52 and 2.20 ± 0.22 days for endometrial cells grown with 10 % PRP and FBS 10 %, respectively.

Endometrial cells cultured with 5 % of PRP, at P1 showed a growth curve with an initial lag phase of 2 days and a subsequent long log phase of 8 days. At P5, endometrial cells demonstrated a growth curve with an initial lag phase similar to that observed for P1, continues with a log phase of 4 days followed by a stationary phase of 4 days. Finally, after 10 days in culture, a death phase was observed. At P10, endometrial cells showed a growth curve with an initial lag phase longer than that registered for endometrial cells at P5 (2-8 days) and subsequent log phase shorter than that observed for P5 (2 days) (Fig. 3b).

#### In vitro effect of PRP on endometrial gene expression

**Fig. 4:**
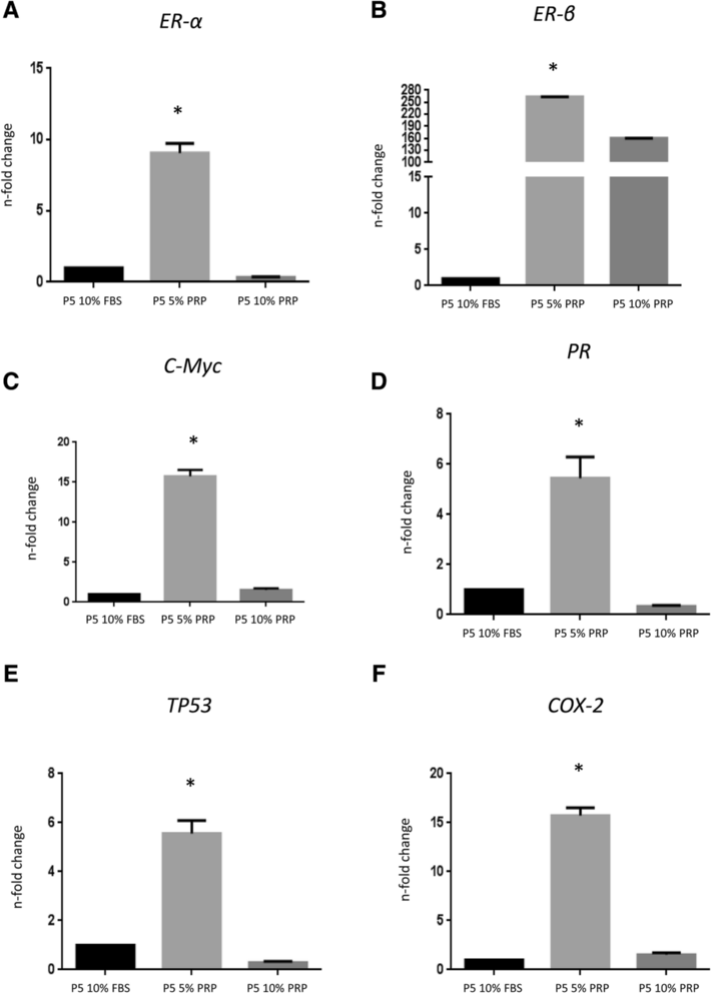
Quantitative PCR analysis for the expression of genes involved in the regulation of oestrus cycle and fetal-maternal interaction like: (**a**, **b**) oestrogen receptors (*ERα* and *ERβ*), (**c**) *c-Myc*, (**d**) progesterone receptor (*PR*), (**e**) tumor protein p53 (*TP53*), and (**f**) prostaglandin-endoperoxide synthase 2 (*COX2*) in cells cultured in presence of 5 % PRP. Also the expression of is reported. Expression levels normalized to the reference gene. Data are represented as fold-change compared with expression observed in cells cultured in presence of 10 % FBS. Values are mean ± SD (*n* = 3). Asterisk depict highly significant (*, *P* < 0.01) differences compared to cells cultured in presence of 10 % FBS

Endometrial cells were cultured in presence of two different concentrations of PRP (5 % and 10 %) until passage 10, and mRNA expression of genes involved in the regulation of oestrous cycle and fetal-maternal interaction was measured by quantitative PCR. PRP 5 % at P5 had a significant effect on the expression of endometrial genes when compared to the expression levels of untreated cells (Fig. 4a-f). The results showed a statistically significant increase (*P* < 0.01) in the expression of all studied genes at P5 in cells cultured in presence of 5 % PRP compared to 10 % PRP or 10 % FBS. In particular, *ER-α* expression increased 9-fold (±0.66), *ER-β* expression increased 260-fold (±6.8), *PR* expression increased 5.44-fold (±0.85), *TP53* expression increased 5.56-fold (±0.52) and *COX2* expression increased 15.74-fold (±0.78). The expression of *c-Myc* increased of 22-fold (±0.24). At P10, a decrease in the expression of all the evaluated genes was observed, with the only exception on TP53, whose expression remains constant.

#### Experiment 3: in vitro effect of PRP after LPS treatment on gene expression

**Fig. 5:**
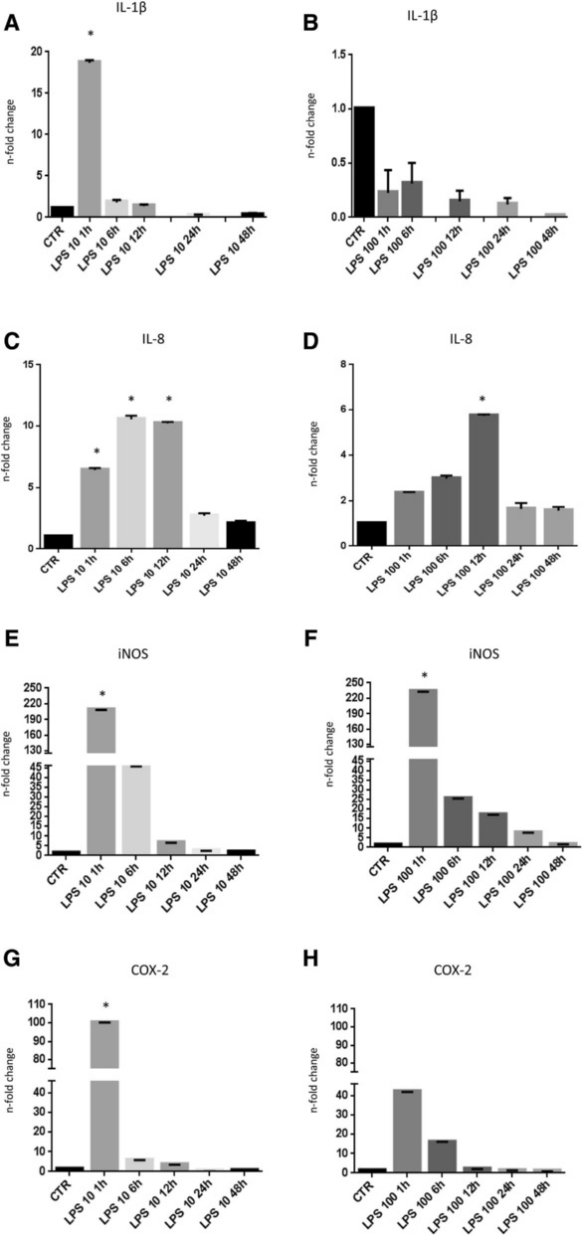
Quantitative PCR analysis for the expression of pro-inflammatory markers such as *IL-1β*, *IL-8*, *iNOS* and *COX2* in cells cultured in presence of 10 (**a**, **c**, **e**, **g**) or 100 ng/ml LPS (**b**, **d**, **f**, **h**). Expression levels normalized to the reference gene *GAPDH*. Data are represented as fold-change compared with expression observed in untreated cells. Values are mean ± SD (*n* = 3). Asterisk depict highly significant (*, *P* < 0.01) differences compared to untreated cells

Endometrial cells at P5 were cultured with or without 10 ng/ml or 100 ng/ml LPS for 1, 6, 12, 24, and 48 h, and mRNA expression of pro-inflammatory genes was evaluated by qPCR. The results are summarized in Fig. 5a,c,e,g for 10 ng/ml and Fig. 5b,d,f,h for 100 ng/ml. Treatment with PRP did not alter *GAPDH* mRNA expression, which was included as a reference gene. The results revealed that the 10 ng/ml concentration of LPS between 1 h and 12 h is the most effective dose to induce an over-expression of pro-inflammatory genes in bovine endometrial cells.

A significant increased (*P* < 0.01) expression of *IL-1β* (18.65 ± 0.32), *iNOS* (208.17 ± 0.18) and *COX2* (99.84 ± 0.31) was observed immediately (1 h) after LPS treatment compared to untreated cells. Expression of *IL-8* was significantly higher (*P* < 0.01) at 6 h and 12 h (10.55 ± 0.29 and 10.22 ± 0.12 respectively) after LPS treatment compared to untreated cells. Within 48 h, all genes returned nearly to baseline.

**Fig. 6:**
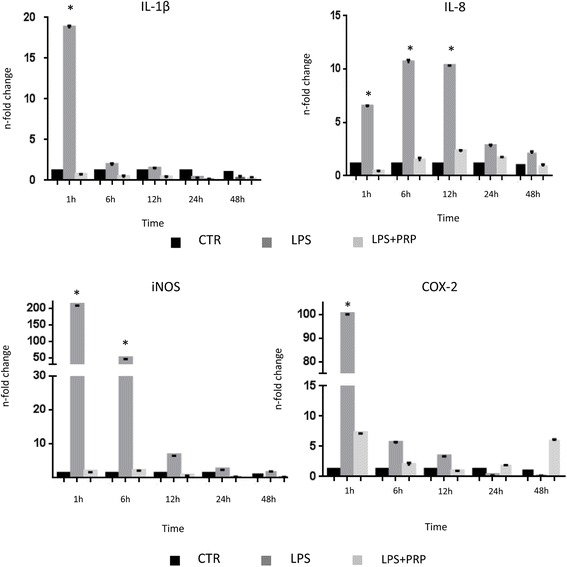
Quantitative PCR analysis for the expression of pro-inflammatory markers such as *IL-1β*, *IL-8*, *iNOS* and *COX2* in cells cultured in presence of 10 ng/ml of LPS and 5 % PRP. Expression levels normalized to the reference gene *GAPDH*. Data are represented as fold-change compared with expression observed in untreated cells (CTR). Values are mean ± SD (*n* = 3). Asterisk depict highly significant (*, *P* < 0.01) differences compared to untreated cells

The PRP treatment had a significant effect (*P* < 0.01) on the endometrial gene expression of these genes when compared to the levels of expression found in the LPS (10 ng/ml) treated cells (Fig. 6a-d). The mRNA expression of the pro-inflammatory cytokine *IL-1β* was found significantly lower compared to untreated cells, passing from 18.65 ± 0.32 to 0.59 ± 0.16 (*P* < 0.01), immediately 1 h after PRP treatment. The mRNA expression of the pro-inflammatory chemokine *IL-8* was significantly lower (*P* < 0.01) with values decreasing at 1 h, 6 h and 12 h after PRP treatment to 0.33 ± 0.14, 1.37 ± 0.31 and 2.23 ± 0.16, respectively, compared to untreated cells. A significant reduction (*P* < 0.01) in the expression levels of *iNOS* (from 208.17 ± 0.18 it fell to 1.66 ± 0.04) and *COX2* (from 99.84 ± 0.31 it fell to 7 ± 0.07) was found compared to untreated cells 1 h after treatment with PRP.

#### Protein release

**Fig. 7:**
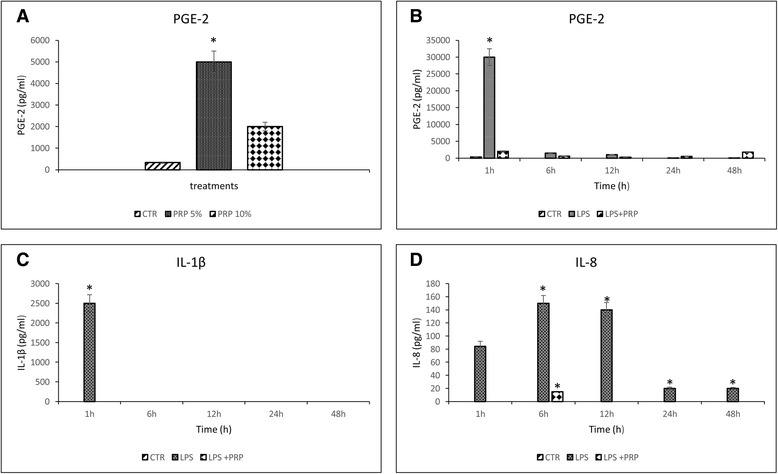
Effect of 5 % PRP at P5 on release of PGE-2 (**a**) and effect of LPS and PRP on release of PGE-2 (**b**), IL-1β (**c**) and IL-8 (**d**) in endometrial cells. Each value represents the mean ± SD of three samples. Values labeled with asterisk are statistically different (*P <* 0.05) compared to untreated cells (CTR)

The results of the release of PGE-2 for endometrial cells treated by PRP at P5 are shown graphically in Fig. 7a. The release of PGE-2, IL-1β and IL-8 by endometrial cells stimulated with 10 ng/ml of LPS and treated with 5 % PRP at different time (1, 6, 12, 24 and 48 h) are shown graphically in Fig. 7 b,c,d). In endometrial cells (CTR), the concentrations of IL-1β and IL-8 were below the limits of detection of the assay. The endometrial cells responded to 5 % of PRP with release of 5000 ± 480 pg/ml of PGE-2 that is statistically different (*P* < 0.05) compared to CTR (330 ± 30 pg/ml). The treatment with 10 ng/ml of LPS induced release of PGE-2 and IL-1β at 1 h (30000 ± 2490 and 2500 ± 220 pg/ml, respectively). The greater release of IL-8 was at 6 h (150 ± 13 ng/ml).

The PRP was able to counteract the action of LPS and significantly (*P* < 0.05) decreased the production of PGE-2, IL-1β and IL-8 mainly between the first and the 24^a^ h.

## Discussion

In the present study, the effect of PRP has been investigated *in vivo* on healthy animals (without endometrial infection) and in an *in vitro* model where bovine endometrial cells were stressed with LPS. After *in vivo* application, our results delineate a trend showing that, *in vivo*, PR expression was detected with a similar average score in all the animals at T0. At T1 in epithelial cells, PR average scores in control and PRP animals remain unchanged. In glandular epithelium and smooth muscle cells, at T1, PR expression increases in PRP animals. These results have to be further explored but may indicate that PRP might be helpful in maintaining and/or increasing the number of progesterone receptors in uterine tissues. In our *in vitro* experiment, bovine endometrial cells were cultured with two different concentrations of PRP (5 % and 10 %) and mRNA levels of different genes were quantified and compared to control cells, cultured with 10 % FBS only. Various research groups have studied the use of PRP as a supplement of cell culture [43–45] demonstrating that PRP increases cell proliferation. PRP confirmed this effect also on endometrial cells, mainly at the concentration of 5 %. This concentration of PRP sustained the shortest population doubling time at P5 while the highest concentration (10 %) inhibited proliferation rate, leading to speculate that this effect may be due to an excess of growth factors. This data was confirmed by growth curve study with 5 % of PRP at different passages. According to this, at P5, 5 % of PRP induced an intensive endometrial cells proliferation. Regarding gene expression, the presence of 5 % of PRP at P5 induced also an up-regulation of the expression of *COX2*, *TP53*, *ER-α*, *ER-β* and *PR*. To test the release of some proteins, we evaluated the secretion of prostaglandin E2 (PGE2). Indeed, it is known that in the bovine uterus, COX2 converts arachidonic acid to prostaglandin H2, which is then converted, by PGE synthase and PGF synthase, to PGE-2 [46]. In these cells, PGE-2 production was modulated in parallel with *COX2* expression in all conditions. This is the first study reporting the effects of PRP on the proliferation and gene expression of bovine endometrial cells *in vitro* and further studies are required to elucidate the mechanisms through which the PRP up-regulates these genes. Although, the molecular mechanisms involved in this up-regulation are not clear, is conceivable that PRP-derived growth factors play an important role. Indeed, the expression of *c-Myc*, that was up-regulated in this study and which is involved in cell proliferation and growth, is activated by EGF that is a component of PRP [29].

In our study, we even further tested the hypothesis that PRP has anti-inflammatory properties and is able to influence gene expression of pro-inflammatory factors in an *in vitro* model of inflammation. To develop an *in vitro* cell culture system with stressed cells and to study gene response, endometrial cells were exposed to the bacterial endotoxin LPS before and after the treatment with PRP. Exposure of endometrial cells to 10 ng/ml LPS resulted in marked changes in the expression of pro-inflammatory genes that was reduced by the addition of 5 % of PRP. The presence of PRP ameliorated the LPS-induced molecular changes in endometrial cells as they increased the levels of gene expression of *IL-1β*, *IL-8*, *iNOS* and *COX2* expression. In parallel with gene expression, the release of some proteins was studied confirming the observations regarding gene regulations. LPS demonstrates to be capable of inducing the release of PGE-2, IL-1β and IL-8 whose pick were obtained 1 h and 12-24 h, after stimulation with LPS. PRP reduce the release of these proteins-and the maximum modulatory activity was observed immediately after 1 h for PGE-2 and IL-1β, and between 12 h and 24 h for IL-8. Therefore, our results indicated consistent anti-inflammatory effect of PRP.

How can we explain these results? Recent literature demonstrates the healing potential of autologous platelet-rich plasma in human medicine, including maxillofacial surgery, dental implant surgery, orthopedic surgery, bone reconstruction, muscle and tendon repair, reversal of skin ulcers, retinal hole repair in eye surgery, and cosmetic surgery [29]. In veterinary medicine, little clinical information exists about its application. PRP is mainly used for equine tendon repair [47] although its application has also been reported for intestinal wound healing in pigs [48] and for the treatment of a large cutaneous lesion in a dog [49]. Recently, our group has proposed its use as an alternative therapy for bovine mastitis and for repeat breeder cows syndrome, showing promising results [32, 50]. Indeed, in acute mastitis, the treatment with only PRP did not show statistically significant differences compared to antibiotic alone or treatment with PRP combined to antibiotic, but it was better than the use of only antibiotic for chronic mastitis [32]. In repeat breeder treatment, after intrauterine administration of PRP the percentage of pregnant cows was 70 % compared to control group, treated with physiological solution, where the rate was 33.33 %. Moreover, immunohistochemical study, by the nuclear antigen Ki-67, a marker of cell proliferation, showed more proliferating nuclei in the treated uterine horn, after three days of administration, compared to the control one [50]. In tissues with a high proliferative rate, Ki-67 is expressed in all cell cycle phases except for the resting or G0 phases [51, 52] and reaches a maximum level during G2 and M [53]. Since Ki-67 was significantly more expressed in uterine horns treated with PRP, Lange-Consiglio et al. [50] supposed that the growth factors released by platelets in the PRP might have had an effect on endometrial cell proliferation. Indeed, in all treated horns there were statistically more proliferating nuclei compared to the controls. Also in our *in vitro* study, PRP induce cell proliferation confirming these previous results [50], but a longer *in vitro* culture time (P5) is necessary to stimulate a good proliferation rate. It is difficult to explain the different times of PRP efficacy between *in vivo* and *in vitro* study, probably they are mainly due to the different doses used.

After the study with PRP on repeat breeder cows, our group tried to test the effect of PRP on *in vitro* endometrial stressed cells by LPS. Results showed that LPS provoked a rapid increase in endometrial mRNA expression and release of IL-1β and IL-8. Previous studies have shown that bovine endometrium expresses the TLR-4/CD14/MD-2 receptor complex in both epithelial and stromal cells [17, 18, 54]. This is a key component of the innate immune system, which is necessary to detect LPS. Subsequent activation of TLR4 triggers the production of cytokines, particularly IL-1β and chemokine IL-8 in bovine endometrium. Accordingly, after LPS treatment, we found an immediate rapid rise in *IL-1β* expression, which had already peaked by 1 h, and had been followed by a rise in *IL-8* expression, with a significant up-regulation at 6 h and 12 h. These waves of cytokine gene activation, short for *IL-1β* and prolonged for *IL-8* until 12 h, are supported by data of DeForge and Remick [55] that studied the kinetics of these cytokines in LPS-stimulated human whole blood as an *ex vivo* model of sepsis. LPS also induces an inflammatory response characterized by the increased expression of *iNOS* and *COX2* and stimulates the secretion of PGE, as demonstrated also by Herath et al. [56]. Prostaglandins, produced by the uterine endometrium, have multiple roles in endometrial function [46]. Indeed, they are key regulators of several reproductive events, including estrous cycle, implantation, pregnancy maintenance and parturition. The release of PGE-2 induced by 5 % of PRP could support these events. In addition, PGE has an important role in the mammalian immune response, acting through prostaglandin E receptors 2 and 4 (PTGER2 and PTGER4) to control inflammation [3]. Instead, the intensive LPS-induced PGE secretion by endometrial cells may impair fertility. In fact, this has been suggested as a pathogenic mechanism after uterine bacterial infection, which could result in an extended luteal phase and thus contribute to an impaired reproductive performance. According to this, *COX2* was greatly up-regulated in the present study by LPS. Regarding the inducible iNOS, it is the main isoenzyme governing the production of NO, and it is considered an important mediator of cellular interactions and a potent regulator of many local processes [57]. iNOS is typically expressed at the sites of inflammation, produces large amounts of NO for a prolonged time and is usually related to the activation of pro-inflammatory macrophages [58]. In this context, our findings showed a significantly higher expression of *iNOS* in cells cultured in presence of LPS. This gene returned nearly to baseline within 48 h, supporting the idea that the inflammatory process is controlled by negative feedback regulation in order to eliminate excessive tissue inflammation and damage [59].

The treatment with 5 % of PRP, after LPS action, down-regulated the expression of all pro-inflammatory genes. The inhibition of NF-kB (nuclear factor kappa-light-chain-enhancer of activated B cells) activation could be a possible mechanism through which PRP exerted these anti-inflammatory effects. Within mammalian cells, including endometrial cells, NF-kB is present in an inactive status [60]. It regulates more than 150 genes, including those involved in inflammation and other immune responses [61]. Upon phosphorylation, it translocates into the nucleus [62] by the interaction of specific receptors on the surface of a cell, and it triggers the inflammatory cascade. In particular, LPS is reported to bind the surface antigen CD44 and induce the translocation of the phosphorylated Nf-kB to the nuclei leading to the increase of pro-inflammatory gene levels [63]. Numerous studies demonstrated that PRP inhibited the translocation of NF-kB to the nucleus [31, 64]. Between different growth factors, indeed, the HGF is described to disrupt the transactivacting activity of NF-kB, with its retention in the cytosol [31].

Based on these results, it would be interesting to explore the mechanisms through which the PRP induces up-regulation of *COX2*, *TP53*, *ER-α*, *ER-β* and *PR* genes and to perform *in vivo* investigation on the potential application of PRP in the treatment of bovine endometritis. Indeed, considering the results obtained in our *in vivo* and *in vitro* studies, it might be possible to envisage treatment of endometritis with PRP combined with administration of exogenous P4. This combination, using intravaginal P4 devices, may improve the fertility in bovine with inadequate circulating P4 concentrations. In this way, at the same time, it would be possible to increase circulating P4 concentration and endometrial PR.

## Conclusions

Although this study is performed on an *in vitro* model, our data indicates that PRP have strong effects on endometrial cells determining high proliferation rate and up-regulation of genes that play an important role in reproduction. The mechanisms through which the PRP up-regulates these genes is not yet clear, but it is conceivable that its growth factors are involved in these mechanisms. The results of the current investigation demonstrated that PRP is capable of effectively decrease gene expression of pro-inflammatory factors *IL-1β*, *I-L8*, *COX2* and *iNOS* after stimulation by LPS, prospecting its possible use in regenerative therapy in *in vivo* endometritis.

## Abbreviations

c-Myc: v-myc avian myelocytomatosis viral oncogene homolog
DT: Doubling time
EGF: Epidermal growth factor
ERβ: Oestrogen receptor β
ERα: oestrogen receptor α
FBS: Fetal bovine serum
HG-DMEM: High glucose-Dulbecco’s modified Eagle medium
iNOS: Inducible nitric oxide synthase
IL-1β: Interleukin 1 beta
IL-8: Interleukin 8
LPS: Lipopolysaccharide
P: Passage
PDGFs: Platelet-derived growth factors
PGE2: Prostaglandin E2
PGF2α: Prostaglandin F2α
PR: Progesterone receptor
PPP: Poor platelet plasma
PRP: Platelet-rich plasma
PTGS2 (o COX2): Prostaglandin-endoperoxide synthase 2
RT-PCR: Reverse transcriptase-polymerase chain reaction
TGF-β: Transforming growth factor β
TLRs: Toll-like receptor
TP53: Tumor protein p53 inducible protein3
VEGF: Vascular endothelial growth factor
